# Recent Advances in Starch-Based Blends and Composites for Bioplastics Applications

**DOI:** 10.3390/polym14214557

**Published:** 2022-10-27

**Authors:** Shishanthi Jayarathna, Mariette Andersson, Roger Andersson

**Affiliations:** 1 Department of Molecular Sciences, Swedish University of Agricultural Sciences, Box 7015, SE-750 07 Uppsala, Sweden; 2 Department of Plant Breeding, Swedish University of Agricultural Sciences, P.O. Box 190, SE-234 22 Lomma, Sweden

**Keywords:** starch, bioplastics, plasticizer, filler, thermoplastic, polymer, blend, composite

## Abstract

Environmental pollution by synthetic polymers is a global problem and investigating substitutes for synthetic polymers is a major research area. Starch can be used in formulating bioplastic materials, mainly as blends or composites with other polymers. The major drawbacks of using starch in such applications are water sensitivity and poor mechanical properties. Attempts have been made to improve the mechanical properties of starch-based blends and composites, by e.g., starch modification or plasticization, matrix reinforcement, and polymer blending. Polymer blending can bring synergetic benefits to blends and composites, but necessary precautions must be taken to ensure the compatibility of hydrophobic polymers and hydrophilic starch. Genetic engineering offers new possibilities to modify starch *inplanta* in a manner favorable for bioplastics applications, while the incorporation of antibacterial and/or antioxidant agents into starch-based food packaging materials brings additional advantages. In conclusion, starch is a promising material for bioplastic production, with great potential for further improvements. This review summarizes the recent advances in starch-based blends and composites and highlights the potential strategies for overcoming the major drawbacks of using starch in bioplastics applications.

## 1. Introduction

Polymers comprise a wide group of materials in which many small molecules (monomers) are linked together to form long chains. Polymers are used in a wide range of applications. Natural polymers have been used for thousands of years, although to a very limited extent in early times, e.g., the use of plant gums as a wood adhesive in house construction or as waterproof material in boat making. The first synthetic polymer, a thermosetting phenol-formaldehyde resin called Bakelite, was invented in 1907 by Leo Hendrik Baekeland [1]. Scientific and technical advances and huge investments in petrochemistry during the second half of the 20th century enabled the development of a range of synthetic polymers for use in a variety of applications [2]. Major petroleum-based commodity polymers currently in widespread use include polyethylene (PE), polypropylene (PP), polystyrene (PS), and polyvinyl chloride (PVC), which are generally called plastics. Total plastics production has increased tremendously in recent decades, reaching close to 460 million tons by 2019 [3,4].

Petroleum-based plastics are not environmentally friendly, since it takes hundreds of years for them to degrade into harmless end-products [1], and petroleum is a finite resource, so sustainable substitutes for plastics are required. The use of synthetic polymers is associated with several drawbacks such as contributing to environmental pollution, high costs of production, and consumption of finite resources. If current trends in plastic production and waste handling continue, it is predicted that around 12,000 Mt of plastic waste will be dumped in landfill or the natural environment by 2050 [5]. Within total plastic production, packaging accounted for the largest share, 40.5% in Europe by 2020 [6]. Thus, the utilization of bio-based polymers to generate bioplastics as substitutes for synthetic polymers has received much attention in recent years in packaging applications [7].

**Figure 1:**
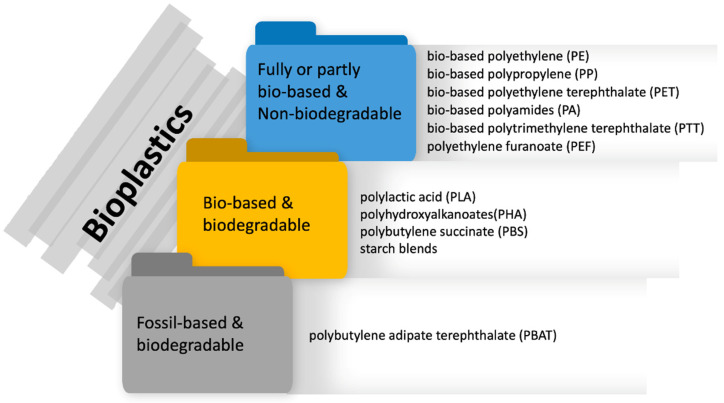
Different types of bioplastics (Source: [8], the illustration is created by the author).

Bioplastics must comply with the requirement of either being produced from bio-based raw material or exhibiting biodegradability or possessing both properties. As displayed in Figure 1, bioplastics materials can be divided into three main categories: (i) bio-based or partly bio-based non-biodegradable plastics (e.g., bio-based PE, PP, polyethylene terephthalate (PET); polyamides (PA), polytrimethylene terephthalate (PTT), polyethylene furanoate (PEF); (ii) both bio-based and biodegradable plastics (e.g., polylactic acid (PLA), polyhydroxyalkanoates (PHA), polybutylene succinate (PBS); different starch blends and (iii) fossil-based biodegradable plastics (e.g., polybutylene adipate terephthalate (PBAT) [8]. Bioplastics currently represent less than 1% of total global plastic production of more than 367 million tonnes per annum [6,8]. However, the demand for bioplastics is growing and they are used in a variety of applications, e.g., packaging, consumer goods, electronics, textiles, automotive, agriculture, construction, etc. The majority of applications are within the packaging sector, which in 2021 accounted for approximately 48% of the total bioplastics market [8].

The use of bio-based polymers instead of synthetic polymers has several advantages, e.g., they are biodegradable, non-toxic, and renewable, and are therefore a good substitute for commodity plastics. Starch has proven to be a promising bio-based polymer that can be used in bioplastics applications and compared to other biopolymers the starch market has a long history [9]. According to the categorization of the bioplastics [8], starch belongs to category (ii). Starch is the major form of carbohydrates in higher plants and is stored in the form of granules with a diameter ranging from <1 μm to 100 μm. These granules differ in shape (e.g., spherical, oval, polygonal, disk-like (lenticular), elongated, or kidney-shaped) depending on the botanical source [10]. Starch is composed of two major macromolecular components, namely amylose, which is predominantly a linear (1→4)-linked α-glucan, and amylopectin, which is a (1→4)-linked α-glucan with (1→6) branch points [11]. Normal starch contains around 20–30% amylose and 70–80% amylopectin [10].

Amylopectin has around 5% branch points, which means that its properties differ from those of amylose [11].

Starch is a major source of energy in the human diet and has various non-food applications, e.g., in the textile industry as a sizing agent and in the paper industry as an adhesive and binder. There has been a considerable amount of research and development on starch-based materials in recent years, with the aim of utilizing starch for bioplastics applications, due to environmental concerns regarding the use of fossil fuel-based plastics. Starch plays a key role in the field of bioplastics, mainly due to its abundance, renewability, biodegradability, and low permeability to oxygen, in addition to being an inexpensive raw material [12]. In 2021, starch blends accounted for 16.4% of total global bioplastics production [8], and in the year 2014, Novamont (Novara, Italy) was reported as the major starch bioplastics producer with the trade name Mater-Bi [9]. 

**Figure 2:**
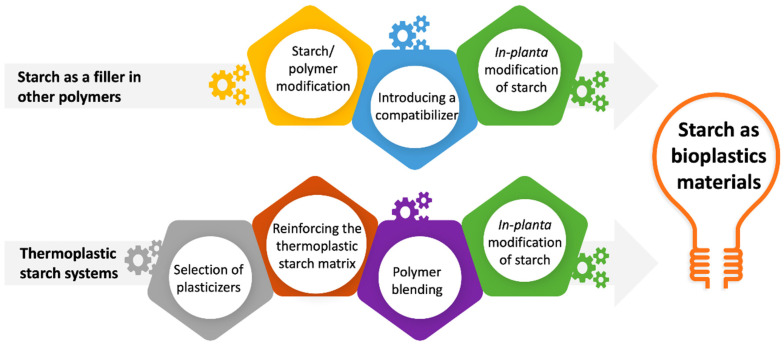
Use of starch in bioplastics applications and strategies to improve the properties of starch-based bioplastics materials.

Composite materials with unique properties can also be created using starch, by incorporating various petroleum-based polymers and other biopolymers into blends with starch. In early development in the 1970s, starch was used as an additive to synthetic polymers to improve biodegradability and exploit the potential of this abundantly available natural resource [13]. However, the use of starch in its native forms in bioplastics applications is limited by poor material properties and water sensitivity [14]. Hence, various approaches to mitigate these issues have been tested over recent years [15]. This article reviews recent research and development on starch-based blends and composites in bioplastics applications both starch as fillers as the main component in thermoplastic starch (TPS) systems (Figure 2) Additionally, the results of attempts to overcome the associated limitations, particularly pertaining to their mechanical and barrier properties are addressed. Mainly focusing on starch as bioplastics material, this review contributes to the biopolymer scientific society together with a few comprehensive reviews that have the main focus on starch for bioplastics applications.

## 2. Starch as Fillers for Other Polymers

Starch is a suitable filler material because of its thermal stability and limited interference with the melt flow properties of most synthetic plastic materials [12]. Blending starch with synthetic polymers increases their biodegradability since starch is naturally degraded by microorganisms leaving a skeleton of synthetic polymers, facilitating their degradability by natural means such as thermal oxidation and ultraviolet photo-degradation [16]. 

The properties of filled polymer materials depend on the size and shape of the filler and its compatibility with the polymer matrix [17]. When starch is used as a filler in materials, particle size plays a major role. A study showed that tensile strength and yield strength of starch-filled thin films made of linear low-density polyethylene (LLDPE) have a negative correlation with the mean granule size of the starch, while starch granule size is linearly correlated with film thickness and light transmittance [18]. 

Since the degree of compatibility between the filler and the polymer matrix plays a major role in determining the properties of filled polymer materials, much research focus should be directed toward investigating ways to improve the compatibility of starch with synthetic polymers. The hydrophilic nature of starch and the hydrophobic nature of synthetic polymers make them incompatible, by hindering the formation of strong interfacial hydrogen bonds between synthetic polymer and starch. Hence, modification of either the starch or the polymer is needed to improve the compatibility. Otherwise, lack of compatibility results in poor adhesion, making the matrix incapable of distributing loading forces equally, leading to a “pull-off” of starch from the polymer matrix upon application of force [16]. This section considers the use of starch, its potentials, and its limitations as a filler material for plastic, using a nonpolar synthetic polymer (LPDE), a polar synthetic polymer (polyvinyl alcohol, PVOH), and a biodegradable polymer (PHA) as examples.

The use of starch as a filler for LDPE has received much attention, but the immiscibility of starch with LDPE limits its applications. A study characterizing LDPE-corn starch blend films with various concentrations of starch ranging from 2.5% to 50% reported weakening of tensile properties, such as tensile strength and elongation at break, with increasing starch content, an effect attributed to incompatibility of starch and LDPE [19]. This was also observed in a study testing starch of different origins (e.g., sago, corn, potato, tapioca, wheat), which reported weakening of material properties of LDPE-starch blends with increasing addition of starch [20]. However, the different blends tested in that study did not show significant differences in physical and mechanical properties depending on the type of starch [20] These results imply that there is a limit on the level of starch that can be included in synthetic polymer/starch blends. 

Another limitation of using starch as a filler material is the poor barrier properties of the resulting polymer-starch blends. The incorporation of starch causes a reduction in intermolecular attractive forces between the synthetic polymer layers, which leads to a large amount of void content in the polymer-starch blend, compromising the barrier properties. Poor barrier properties for water vapor are a common problem associated with starch blends and are primarily due to the hydrophilic nature of the starch. One study reported an increase in water vapor permeability with increasing starch content in an LDPE/starch composite, with a more significant increase in water vapor permeability when the starch content was over 30% [19]. An increase in oxygen permeability with increasing starch content was also observed in that study, with a significant increase when the starch content was over 20%. Moreover, the grease resistance of LDPE-starch films decreased with increasing starch content, with 10 days of resistance to grease in plain LDPE films decreasing to 3 days when 50% LDPE was replaced with starch [19]. 

PVOH has been identified as a more compatible material with starch than LDPE, due to the polar nature of PVOH. Starch and PVOH are compatible since the functional hydroxyl groups of these two materials form hydrogen bonds that keep the materials tightly linked to each other [16]. Incorporation of starch into PVOH improves the biodegradability of PVOH films and increasing the starch content in the blend further enhances the biodegradability [21,22]. One study observed improved mechanical properties of PVOH films when starch was incorporated up to a level of 10%, with both tensile strength and percentage elongation, an effect attributed to hydrogen bond formation between PVOH and starch [22]. However, another study found that both tensile strength and elongation at break were reduced in PVOH-starch films when the starch content was increased and suggested that higher starch loadings may lead to the formation of more pronounced filler-filler interactions than filler-matrix interactions, weakening the tensile strength of the films [21]. It was also found that the incorporation of starch led to higher water uptake by the PVOH/starch blend films compared with pure PVOH films, an effect attributed to the hydrophilic nature of the starch [21]. 

The use of starch as a filler in biodegradable polymers has received much research attention in recent years since it can help in producing fully biodegradable polymer/starch blends and composites. Particularly, starch-filled PHA materials have been studied extensively, because although PHA is a fully biodegradable polymer, its usage is limited due to the high cost. Hence, blending PHA with starch filler is an economically feasible solution. Other than the economic advantage, starch serves as a reinforcing filler, contributing to improved mechanical properties while producing a fully biodegradable blend [23]. It has been found that when starch is incorporated as a filler with poly(hydroxybutyrate-co-valerate (PHBV), which is a copolymer of PHA, starch degrades faster than PHBV and accelerates the degradation of PHBV [24]. A detailed discussion on starch-PHA blends is provided in Section 4.3 of this review.

### Approaches to Improve the Performance of Starch-Filled Polymers

Various attempts have been made to improve the performance of starch-filled polymer blends and composites, with a focus on improving the compatibility of starch and other polymers. This has been attempted through the modification of starch or the other polymer. Various approaches to improve the miscibility between starch and other polymer components have been extensively reviewed [16]. Introducing a compatibilizer, which is usually a functionalized polymer, is one of the most common approaches. A compatibilizer can form covalent bonds between the synthetic polymer and starch to improve the interfacial interaction between the two phases [25]. Several types of compatibilizer have been tested, e.g., maleic anhydride [26] and maleate esters such as dibutyl maleate (DBM) [27], to yield better mechanical properties that can be attributed to improved interfacial adhesion between the hydrophilic starch and hydrophobic polymer. 

Modifying the starch component to impart hydrophobic characteristics is another approach used to improve the compatibility of starch and synthetic polymers. In conjunction with this approach, converting starch into a hydrophobic derivative by phthalation is common practice. For example, starch-phthalate-filled LDPE blends are reported to exhibit better adhesion, resulting in better mechanical properties compared with unmodified LDPE/starch blends, and also better degradation in soil [28]. In an alternative approach, Rivero et al. [29] made starch amphiphilic by microwave-assisted esterification with octenyl succinic anhydride to produce modified starch and tested the use of the amphiphilic starch as a compatibilizer for cassava starch-filled LLDPE blend. They observed better mechanical properties of the blend when octenyl succinic anhydride-modified starch was employed as a compatibilizer, with yield stress values that were almost similar to those of pure LLDPE, especially at lower starch inclusion rates [29].

A recent study reported using acid hydrolysis of rice and potato starch to produce starch nanocrystals to add to the LDPE matrix. LDPE/nano starch blend displayed better compatibility and promising properties in films. The incorporation of 1% rise starch nanocrystals could reduce the oxygen permeability of films while increasing the thermal stability. The incorporation of 1% potato starch displayed an improved modulus of elasticity than LDPE films. The addition of nanocrystals of both types increases the hydrophobicity of the films, and elongation in the Longitudinal direction but made the films opaquer than LDPE films [30]. 

Physical and/or chemical cross-linking of starch and/or polymer blends has also been proven to be effective in improving the properties of starch-filled polymer blends. For example, a study examining the influence of trimethylolpropane triacrylate as a cross-linking agent and electron beam irradiation of sago starch before incorporation into an LDPE polymer found that these modifications effectively altered the mechanical, thermal, and degradation properties of the polymer [31]. In that study, Young’s modulus increased, while ductility and melting temperature decreased, with increasing trimethylolpropane triacrylate concentration and electron beam irradiation dose [31]. Another study reported improvement in water resistance, thermal stability, and mechanical properties of PVOH/Starch bio blend films by the addition of citric acid as a cross-linker and glycerol as a plasticizer. The thermal and mechanical properties of PVOH/Starch/Citric acid/Glycerol bioblend film were comparable to commercial LDPE and PP films [32].

Epichlorohydrin (ECH) has been used as a crosslinking agent in fossil fuel based-polymer/starch blends. A study reported improvement in tensile strength, percent elongation and strain energy of the ECH crosslinked starch-filled LDPE films compared to the native starch-filled LDPE films [33]. ECH crosslinking of hydrolyzed starch-g-PAN (HSPAN)/PVOH blend films was tested effective in overcoming the phase separation of blend films by improving the compatibility between two polymers due to the crosslinking reaction with Epichlorohydrin between the hydroxyl groups of starch and PVOH. Crosslinking of blend films could reduce the hydrophilicity of HSPAN/PVOH films. Moreover, crosslinking reaction with ECH could improve the mechanical properties of the (HSPAN)/PVOH blend films and the improvement of the tensile strength and strain-at-break were proportional to the content of ECH [34]. 

Other than the chemical methods of starch modification, physical and enzymatic methods of starch modification exist, and enzymatic methods are minimally in use due to their complexity and time-consuming nature. Physical modification methods are preferred as environmentally friendly and safe methods of starch modification. Gamma irradiation is one of the methods to physically modify the starch [35]. Gamma irradiation modification of various polymer/starch blends are reported [32,36]. Irradiation leads to crosslinking, and main-chain scission of biopolymers. Gamma irradiation up to 15 kGy could improve the thermal stability of LDP/natural rubber/PVOH/starch/glycerol blend and tensile strength and elongation were improved up to 30 kGy. However, irradiation dose increment up to 30 and 40 kGy negatively affected the thermal stability and mechanical properties respectively [36]. Improvement in the thermal stability and mechanical properties as influenced by gamma irradiation (at a dose of 10 kGy) was reported also for PVOH/starch/citric acid/glycerol bio blend films [32].

## 3. Recent Advances in TPS Systems

Plasticized or destructured starch, called TPS, is produced by the formation of hydrogen bonds between the hydroxyl groups of starch and molecules such as water, glycerol, and sorbitol, which are generally referred to as “plasticizers”. In the presence of plasticizers and at high temperatures (90–180 °C) and shear, starch readily melts and flows, providing the possibility for its use in injection, extrusion, or blowing production processes, similar to those used for synthetic thermoplastic polymers [37]. There are various applications of TPS, such as food packaging, disposable eating utensils, trash bags, compostable films, and bags for agriculture and retail. 

There are two methods of producing TPS, the casting solution method, and the extrusion process. The casting solution method is mostly used for experimental purposes in laboratories, while in large-scale industrial production the extrusion process is the preferred method [38]. The casting method produces more homogeneous films than those produced by the extrusion method, and hence casting films have lower opacity [39]. Moreover, casting films are reported to have lower water vapor permeability and are superior in terms of stress at break than films produced by extrusion [39].

However, TPS has some disadvantages, such as a tendency to retrograde with time, poor water resistance, and unsatisfactory mechanical properties, particularly in wet or dry environments. To address those drawbacks of simple TPS, several approaches are used in practice, e.g., selecting different sources of starch, using different kinds of plasticizers, blending TPS with other polymers (natural and synthetic), and using fillers or reinforcing materials. Recent advances in various approaches to improve the properties of TPS systems are discussed in this section.

### 3.1. Selection of Plasticizers

The plasticizers commonly used to produce TPS include polyols such as glycol, sorbitol, ethylene glycol, glycerol, and sugars. However, simple TPS made by only plasticizing starch tends to retrograde with time and this retrogradation embrittles the material [40]. To achieve better material properties, it is critical to prevent retrogradation. Many studies have shown the effectiveness of using several plasticizers in combination as an approach to hinder retrogradation and produce TPS with promising properties for various applications. For example, Krogars et al. [41] observed better stability of starch films plasticized with a combination of sorbitol and glycerol (1:1). They found that using a combination of plasticizers prevented migration of plasticizers out of the starch film, suggesting tighter binding of plasticizers within the film due to induced interactions when plasticizers of two different sizes are used, as opposed to using each plasticizer alone. They observed no crystallization of the film, which otherwise leads to film brittleness [41]. Thus, problems with the use of sorbitol as the sole plasticizer due to its tendency to migrate out of the film and result in recrystallization over time could be solved by using plasticizers in combination.

Plasticizers containing amide groups, such as formamide, acetamide, and urea, have been used as another way of preparing retrogradation-resistant TPS. A study testing formamide as a novel plasticizer found an ability to form more stable hydrogen bonds with hydroxyl groups of starch [42]. Formamide was effective in retarding retrogradation and improving the flexibility of a film made of corn starch in that study, although the tensile strength and Young’s modulus of the formamide-plasticized starch films were lower than those of glycerol-plasticized starch films. However, elongation at break and energy break were higher in formamide-plasticized starch films [42]. 

Another study found that formamide (10 wt%) and urea (20 wt%) together make a more effective novel plasticizer mix to produce TPS with good mechanical properties (in terms of tensile stress, strain, and energy at break), high thermal stability, and better retrogradation resistance than TPS plasticized with glycerol (30 wt%) [43]. It also found that formamide and urea effectively hindered retrogradation of TPS, properties the authors attributed to the formation of new, stable, and stronger hydrogen bonds by both urea and formamide with starch, and to the fact that formamide is a good solvent for urea, which allows both formamide and urea to exist in their molecular forms in TPS. The study also showed that TPS plasticized by urea (20 wt%) and formamide (10 wt%) was characterized by better water resistance than TPS plasticized by glycerol [43].

A study comparing TPS made using a combination of ethylene bisformamide and sorbitol with TPS made using single plasticizers (glycerol, ethylene bisformamide, or sorbitol) observed superior performance of the TPS made using a combination of ethylene bisformamide and sorbitol, which exhibited better tensile stress, elongation at break, water resistance, and thermal stability [44]. 

The use of urea and ethanolamine as plasticizers for TPS was examined in another study [45]. It found that a mixture of urea and ethanolamine formed more stable and stronger hydrogen bonds with starch molecules than glycerol and that ethanolamine was a good solvent for urea. As a result, urea and ethanolamine plasticized corn starch-based TPS showed better thermal stability and mechanical properties, and suppressed retrogradation, compared with the conventional glycerol plasticized TPS [45].

Aliphatic amidediol as a candidate plasticizer has been used in combination with glycerol to produce TPS from corn starch [46]. That study found that the mixed plasticizer formed more stable and stronger hydrogen bonds with starch, with better mechanical properties in terms of tensile stress and elongation at break compared with the glycerol-plasticized TPS. Moreover, the TPS with mixed plasticizer exhibited better water resistance than the glycerol-plasticized TPS [46].

Hence, amide groups containing plasticizers used in combination with other plasticizers have been proven to be promising for TPS production.

### 3.2. Reinforcing the TPS Matrix

Reinforcing the TPS matrix is another approach to improving the properties of TPS. Reinforcing helps the TPS to overcome limitations related to low tensile strength, severe deformation, and high hygroscopicity [47]. Different types of fibers derived from lignocellulosic wastes have been tested for use as reinforcing agents for TPS, with some examples shown in Table 1. Natural fibers are an attractive alternative to reinforce polymeric composite matrixes due to the features such as low specific density, low cost, high strength, high sustainability and decreased tool wear [48].

The chemical composition and morphology of the fibers, interfacial adhesion, fiber distribution, and orientation between the fiber and the polymeric matrix result in the varying performance of different types of fibers when incorporated into TPS [49].

Fiber size plays an important role in determining the final properties of TPS [56]. In general, nanofibers incorporated into TPS give better mechanical properties and water resistance than microscale fibers. This is due to the great reinforcing potential of nanocellulosic fibers and their ability to form strong hydrogen bonds with the starch matrix due to their chemical similarity [57,58]. Significantly improved mechanical properties of a TPS matrix were found with kenaf bast cellulosic nanofibers compared with raw fibers (0.5 µm) [57]. In that study, increasing the nanofiber content resulted in improvements in Young’s modulus and tensile strength of the nano fiber-reinforced TPS films, but at the expense of reduced elongation at break. Reduced elongation at break can be attributed to the reduced mobility of starch chains [57].

Corn starch-based biocomposites plasticized with glycerol and reinforced with nano-sized bacterial cellulose have been found to be superior to biocomposites made using micro-sized jute fiber in terms of mechanical properties, thermal stability, water resistance, and optical transparency, improved properties attributed to better interfacial bonding between the matrix and the nano-sized fibers [58]. 

Some other modifications of fiber are reported to have positive impacts on the properties of the TPS matrix, e.g., surface modification of cellulose fiber using air plasma treatment improves the adhesion of fiber to the TPS matrix, resulting in improved mechanical properties of the fiber-modified TPS compared with pure TPS [59]. A study in which starch was modified by cross-reaction with oxidation and plasticization, and sisal fiber was oxidized and treated with urea to produce oxidized ureal fiber, reported good effectiveness of modification of both starch component and fiber component in improving the compatibility of starch and fiber [60]. This was because the incorporation of oxidized ureal fiber into oxidized starch caused the formation of hydrogen bonds between starch and fiber [60]. Another study found that using oxidized starch along with unmodified fiber resulted in the formation of new hydrogen bonds between the plasticizer, oxidizer, and starch [61]. The thermoplastic oxidized starch in that case yielded better mechanical properties by hindering post-crystallization of the TPS matrix [61]. Another recent study showed that using alkaline-treated rice husk in thermoplastic cassava starch biocomposites is more effective in terms of mechanical properties and bio-degradability than using untreated rice hull fibers [62]. The study showed that alkaline-treated rice hull possessed more OH groups than untreated rice hull and was reported to give a higher tensile strength to the TPS biocomposites [62]. As reported by another recent study, simultaneous modification of both the sugarcane bagasse fiber component and starch by acetylation could produce a composite material with good mechanical properties and water resistance [63].

Using nano silicon dioxide (nano-SiO_2_) to reinforce the TPS matrix has been found to yield better results in improving the properties of the matrix [25,64]. The size of nano-SiO_2_ plays an important role in determining the properties of TPS composites [64]. That study reported uniform dispersion when using 100 nm nano-SiO_2_ with potato starch molecules compared with other sizes (15, 30, and 80 nm) in forming potato starch films. The 100 nm nano-SiO_2_ formed strong hydrogen bonds with potato starch, so the water vapor transmission rate was reduced, and film tensile strength increased with the incorporation of nano-SiO_2_. The incorporation of nano-SiO_2_ was also effective in imparting antibacterial activity to the films, particularly against *Escherichia coli*, because nano-SiO_2_ was able to destroy the structure of cell membranes by adsorption to bacterial cell walls [64].

In addition to nano-SiO_2_, many studies have reported the use of nanoclay to reinforce the TPS matrix. One study examined the feasibility of using Cloisite 30B nanoclay in a TPS-polypropylene nanocomposite and found that Cloisite 30B was well dispersed in the polymer matrix and contributed to improving the compatibility of the polymers while improving the stiffness and the biodegradability of the matrix [65]. Other inorganic mineral additives to improve the properties of TPS have also been tested. Among these, montmorillonite [66], kaolin [67], rectorite [68], and calcium carbonate [69] have been shown to improve the mechanical properties and water resistance in TPS composites. Moreover, retrogradation of TPS upon storage is hindered when inorganic mineral materials are used [67], and TPS composites with inorganic minerals have a higher rate of biodegradability than pure TPS [69]. 

A study using starch nanoparticles produced by ultrasound treatment to reinforce a TPS-PBAT composite observed improved mechanical properties of the reinforced composite films in terms of Young’s modulus and elongation at break at 1% incorporation level of starch nanoparticles [70]. Moreover, the films exhibited reduced water vapor permeability and water absorption compared with the films without starch nanoparticles [70]. Thus, that study revealed a new possibility of using starch nanoparticles to reinforce a polymer matrix. 

### 3.3. Polymer Blending

#### 3.3.1. TPS-Biodegradable Fossil Fuel-Based Polymers

Among the attempts made to overcome the poor mechanical properties and water resistance associated with pure TPS, TPS-biodegradable fossil fuel-based polymer blends and composites have gained much attention. With this combination, it is possible to achieve desired properties while retaining the biodegradability of the blends and composites. Examples of fossil fuel-based biodegradable polymers used to blend with starch include PBS [71], PVOH [72], polycaprolactone (PCL) [73], and PBAT [25]. 

A novel approach to using PVOH in formulating TPS/PVOH composites has been reported recently [72]. This approach, which involves using PVOH microspheres as a reinforcing agent in the TPS, permits the composite to contain a very limited amount of PVOH, whereas in other approaches the blends/composites contain at least 40–50% PVOH, leading to high costs [74,75]. In the novel approach, adding 1 wt% PVOH microspheres to the TPS matrix effectively improved the tensile strength, elongation at break, and impact strength compared with pure TPS, and also enhanced the thermal stability of the TPS [72]. 

A problem in formulating TPS-synthetic polymer blends is the poor immiscibility of hydrophobic polymers with hydrophilic TPS. Approaches to overcome this poor compatibility between TPS and biodegradable fossil fuel-based polymers include adding a compatibilizer [71] or compatibilizers in combination [25], reinforcing agents [25], cross-linking agents [76], novel types of plasticizers such as calcium chloride [77], and different levels of plasticizers [73].

#### 3.3.2. TPS-Non-Biodegradable Fossil Fuel-Based Polymers

Formulating starch and non-biodegradable fossil fuel-based polymer blends/composites have been attempted to improve the biodegradability of synthetic polymers, mainly using starch as a filler in native form or as TPS. Recent attempts have focused more on using starch as the main component in polymer blends, where starch is used as TPS. In such blends, when the biodegradable component is present in sufficient amounts, its degradation by microbial action upon disposal contributes to the loss of integrity of the inert synthetic polymer, causing it to disintegrate and slowly disappear [78]. Hence, such blends and composites with a higher starch content would be a more environmentally friendly and economically feasible approach to utilizing starch for bioplastics applications. 

Some common examples of synthetic non-biodegradable polymers that have been tested in formulating TPS/synthetic polymer blends and composites include LDPE [79], high-density polyethylene (HDPE) [26], LLDPE [80], PP [65], and PS [81]. However, weak adhesion and compatibility between hydrophilic starch and the hydrophobic synthetic polymer is a major problem for such composites, imposing limitations on their use due to weak functional properties. Various attempts have been made to improve the compatibility between synthetic polymers and starch, through e.g., the use of compatibilizers [26,80,82] reinforcing materials [65], or a combination of compatibilizers and reinforcing materials [83,84], or modifying the processing conditions [79,85].

## 4. Starch-Bio-Based Polymer Blends and Composites

Starch is used in formulating blends and composites together with bio-based polymers to improve mechanical properties and reduce the associated costs. This section briefly discusses a few examples of starch/bio-based polymer blends and composites. 

### 4.1. Starch-PLA Blends and Composites

PLA is an aliphatic, biodegradable, and hydrophobic polyester produced by lactic acid polymerization. PLA possesses excellent mechanical properties that are comparable to those of synthetic petroleum-based polymers (e.g., PET and PS). However, there are several limitations associated with the use of PLA, such as the high cost of production and its brittle nature [86]. Thus, blending PLA with starch has been tested as a feasible strategy to reduce production costs while enhancing mechanical properties. However, weak interfacial adhesion of hydrophobic PLA and hydrophilic starch results in the production of fragile and brittle composites, and therefore several methods have been tested to improve the interfacial adhesion between PLA and starch. 

One common approach to improve the interfacial adhesion and reduce the phase separation of starch and PLA is to use compatibilizers. Greater interfacial adhesion improves the desired material properties of the blends. The compatibilizers, which act as cross-linking agents that chemically bind PLA and starch molecules together, are grafted onto PLA chains [87] to starch [88,89] or a third component such as PCL [90]. 

Incorporating the third component as a softening agent to impart ductility and flexibility to the matrix has been proven to be effective in starch/bio-based polymer blends. Examples of such components include PBS [91] and PCL [92]. One study reported increasing elongation at the break of the material as the PBS content increased in the TPS-PLA blend [91]. Another study demonstrated that PCL improves the impact and elongation at break properties of PLA [92]. Hence, incorporating PBS and PCL contributes to improving the ductility of PLA-starch blends. 

Incorporating an elastomer is another approach for toughening PLA-starch blends. Incorporating elastomers toughens a material by absorbing energy upon stress, due to the rubbery dispersed phase that forms within the brittle polymer matrix [86]. However, most elastomers require a compatibilizer to improve their compatibility with other polymers in the blend. The use of glycidyl methacrylate grafted poly (ethylene octane) (GPOE) in PLA/TPS blends has been tested [93]. That study demonstrated good compatibility between PLA and TPS, with significant improvement in elongation at break, and impact strength of the GPOE) incorporated PLA/TPS blend compared with the non-GPOE blend. Polyethylene octane (POE) was used in that study as the elastomer to induce toughening of the blend and grafting with glycidyl methacrylate has improved the compatibility of POE with starch and PLA [93]. 

Another way of improving the compatibility of starch and PLA is by starch modification, which is a more economical approach than modification of PLA since starch is usually the minor component in a PLA/starch blend. A common approach for starch modification is to make the starch component hydrophobic by substituting the hydrophilic hydroxyl groups with hydrophobic groups, which improves the interfacial adhesion with the hydrophobic PLA component. Incorporating plant oil derivatives via compatible agents is one of the most promising approaches to impart hydrophobicity to starch. Various attempts have been made to couple starch with different types of plant oils, such as soybean oil [94] cardanol [95], castor oil [96], and tung oil [97] in formulating starch/PLA blends and composites. Another approach to impart hydrophobicity to starch is the acetylation of the starch, which increases the hydrophobicity due to the formation of relatively hydrophobic acetylated starch esters [98]. Acetylation of starch could improve the mechanical properties and thermal stability of starch/PLA blends [99]. 

A recent study reported the effectiveness of a sandwich-architecture film of pea starch and PLA for fruit preservation. The adhesion between pea starch and PLA was improved by the incorporation of an octenyl succinic anhydride-modified pea starch (OMPS) interlayer. Octenyl succinic anhydride esterification of the pea starch made the starch more hydrophobic. The modified starch contained both hydrophilic and hydrophobic functional groups which made it effective in using it as an interlayer for improving the interfacial incompatibility between starch and PLA. The films with sandwich architecture had improved barrier properties for water vapor and oxygen [100].

Some studies report good effectiveness of using reinforcing agents to improve the compatibility between TPS and PLA, e.g., the use of coir fiber to produce a stiff, hard composite with improved compatibility between the TPS and PLA phases was reported [101]. The improved compatibility between TPS and PLA, in that case, is most likely due to the high friction induced during the process due to fiber-fiber collision, which leads to the high viscosity of the TPS/PLA/coir fiber composite compared with the TPS/PLA blend. Another study used unmodified nanoclay as both a reinforcing agent and to improve the compatibility between TPS and PLA [102]. 

A novel method to improve the compatibility of starch and PLA without using external components such as compatibilizers or plasticizers is co-grinding during the production of PLA/starch composites [103]. In that approach, starch-filled PLA composite materials produced by co-grinding in a tumbling ball mill have been found to reduce the hydrophilic nature and polar energy component of starch, enhancing its interactions with the PLA component [103]. 

### 4.2. Starch/Natural Rubber Blends and Composites

A few studies have tested the blending of TPS with natural rubber, e.g., natural rubber from *Hevea brasiliensis* However, phase separation limited using a higher amount of rubber in starch/rubber blends and composites. Phase separation depends on the plasticizer content (glycerol), where glycerol acts as both the plasticizer and starch rubber compatibilizer [104]. 

Various approaches to improve TPS/rubber blends have been reported. One study showed the good effectiveness of rubber modification in formulating thermoplastic starch/natural rubber/clay nanocomposites [105]. Scanning electron microscope (SEM) observations of unmodified rubber/TPS nanocomposites revealed poor interfacial adhesion between the TPS and rubber phase, likely due to the hydrophilic character of the TPS and the hydrophobic character of the rubber. However, finer dispersion and improved interfacial adhesion were observed when hydrophilic polydimethylaminoethyl methacrylate (DMAEMA) grafted modified rubber was used, an effect attributed to the formation of hydrogen bonds between DMAEMA grafted latex particles and starch molecules [105]. Hence, the modification of rubber significantly improved the tensile strength and elastic modulus compared with TPS/unmodified natural rubber blends [105]. Formulation of a TPS/epoxidized natural rubber/chitosan blend was reported in another study, where adding chitosan had positive effects on the tensile properties of the blend because of the reaction between the amino groups of chitosan and the epoxy groups of the epoxidized rubber [106]. 

### 4.3. Starch/PHA Blends and Composites

PHAs are a group of natural biodegradable polyesters of microbial origin. Their applications as a biomaterial have been limited to date due to poor mechanical properties, high cost of production, limited functionality, incompatibility with conventional thermal processing techniques, and susceptibility to thermal degradation. Those poor characteristics of PHAs require various modifications, e.g., blending PHAs with other types of polymers [107]. Blending PHAs and starch is an attractive solution to limitations associated with pure PHAs and pure starch. Since PHAs are hydrophobic and have good film-forming capabilities, blending with starch helps to overcome the limitations of using starch or PHAs as a sole polymer for technical applications. 

Poly-3-hydroxybutyrate (PHB) is the most common category of PHA used to date in formulating blends with starch. PHB has physical properties similar to those of PP, but the high cost of PHB limits its usage. Hence, blending PHB with starch, which is an inexpensive and completely biodegradable polymer, is an interesting approach. The feasibility of blending thermoplastic starch with PHB has been reported [108]. Blending thermoplastic starch up to 30 wt% with PHB improved the tensile strength, Young’s modulus, and extension to break (%) compared to pure PHB while producing a lower-cost material. These PHB/starch blends extend the use of PHB as a coating material on paper or cardboard for food packaging applications [108]. A study assessing the effects of using TPS of different origins, including potato, corn, and soluble potato starch, with different ratios of glycerol in producing TPS/PHB, blends found that blending TPS with PHB improved the mechanical properties in all cases compared with pure TPS [109]. Moreover, TPS produced by potato starch and with a low degree of gelatinization showed significantly improved tensile strength and tear strength, indicating that structural changes of the initial starch type and degree of gelatinization play a vital role in determining the final performances of the blends [109]. It can be concluded that blending PHB and starch improves the properties of both starch and PHB compared with their pure counterparts.

Although blending PHAs with starch is a feasible approach to reduce the cost and improve the mechanical properties, the incompatibility between starch and PHAs prevents the formation of intact films, making the films more brittle. Various attempts have been made to improve the compatibility between the two polymers. A recent study reported the use of poly(vinyl acetate) (PVAc)-modified corn starch to improve the compatibility and flexibility of PHA/starch blends [110]. Grafting cornstarch with hydrophobic PVAc chains was shown to improve the compatibility of starch and PHB and dispensability in the matrix. PHB was well mixed with the PVAc component in the modified corn starch, as revealed by similar glass transition temperature (T_g_) for all the PHB/modified corn starch blends. Additionally, the inclusion of modified corn starch improved the thermal stability of PHB, as demonstrated by higher thermal degradation temperature in the PHA/modified starch blends than in pure PHB [110]. 

A recent study tested the use of various cross-linking agents (citric acid, adipic acid, borax, and boric acid) to improve the mechanical and barrier properties of starch/PHA composite films produced by extrusion blowing [111]. In that study, the starch/PHA blend consisted of 80% starch, whereas in many other studies starch has been included at levels below 50% in blends, as a filler. The results showed that the films with cross-linking agents did not have any obvious phase separation, while phase separation was observed in films without cross-linking agents. The addition of cross-linking agents also significantly improved the mechanical properties of the films in terms of tensile strength and elongation at break compared with films without a cross-linking agent. The formation of strong intermolecular interactions between cross-linking agents, starch, and PHA is likely to be the reason for the improved tensile strength, while the improved elongation at break might be attributable to the more homogeneous dispersion of the components in blends with cross-linking agents. The cross-linked films in that study exhibited lower water vapor permeability than the films without cross-linking agents, which might be primarily due to decreased number of free hydroxyl groups in starch chains. Additionally, the films with cross-linking agents had superior resistance to oxygen permeability than films without cross-linking agents, and the thermal stability and light transmittance were improved due to cross-linking. Individual variations were observed, however, as affected by the type of cross-linker. Citric acid and adipic acid were found to be more suitable for the preparation of starch/PHA composite films than borax and boric acid [111].

A recent study reported in situ grafting of PHAs onto starch as an appropriate method to improve the interfacial adhesion of starch and PHA in an economical and environmentally friendly way, with limited usage of chemicals [112]. In that study, dicumyl peroxide (DCP) was used as a free radical initiator for in situ grafting of PHAs onto the starch. Grafting was found to improve the interfacial adhesion of starch and PHA and lead to improved mechanical properties in terms of tensile strength and Young’s modulus up to a DCP content of 2 wt%. Moreover, the PHAs/starch/DCP blends showed higher thermal stability than the PHA/starch blends.

## 5. Recent Trends in Production of Functionalized Starch-Based Composites

Starch-based composites with antimicrobial and/or antioxidant properties, for food packaging applications, have attracted research interest in recent years. This type of packaging ensures food safety as an additional advantage, apart from biodegradability. It can also eliminate the use of food additives such as food preservatives and antioxidants, making it a viable alternative in terms of production economics, sensory properties of the food, and consumer health. This section focuses on some recent advances in producing novel, functionalized starch-based composites.

Successful incorporation of antimicrobial agents into food packaging materials has been reported in recent years. Several types of antimicrobial agents have been incorporated into starch-based composites, as presented in Table 2.

Another recent advance in the development of starch-based food packaging materials is the incorporation of antioxidants into starch-based packaging materials that have the capability for delaying food spoilage due to oxidation. In this approach, antioxidants are added to the packaging material, as opposed to adding them directly to the food in high doses. Several studies have examined the use of antioxidants in food packaging (Table 2). Some studies have also tested the incorporation of both antimicrobial and antioxidant properties into starch-based films (Table 2).

The development of intelligent packaging is another recent advance in producing functionalized starch-based composites in the food packaging sector. Intelligent packaging carries out special functions such as tracking, sensing, and communicating, which can influence consumer decision-making, and this novel approach can be used to enhance food safety and quality [113]. For example, one study tested incorporating anthocyanins from jaboticaba flour as a pH indicator in biofilms made from pinhão (*Araucaria angustifolia*) starch and found a visual color change in the anthocyanin incorporated biofilms when immersed in solutions with different pH values, indicating good potential for using the material as a pH indicator in starch-based food packaging [114]. 

**Table 2:** Examples of different types of functionalized starch-based composites.

Target Property	Example	Important Results	Reference
Antimicrobial action	Chitosan in PLA/starch blend	PLA and starch were a slow-release matrix of chitosan. Chitosan release was fast at the beginning and slowed down later which imparted long residual antimicrobial properties to the blend. Showed effectiveness in preserving food with high water activity such as fresh meat.	[115]
	Peel particles of pomegranate in starch-based films	Pomegranate peel acted both as an antimicrobial agent and a reinforcing agent. The films showed effectiveness for both gram-positive (*S. aureus*) and gram-negative (Salmonella) bacteria. The films showed better mechanical properties (Young’s modulus, tensile strength, stiffness) due to the reinforcing action of the pomegranate peel particles	[116]
	Essential oils from oregano (e.g., carvacrol and citral) in sago starch and guar gum-based films	The films showed antimicrobial activity against Bacillus cereus and *Escherichia coli*. Essential oils acted as a plasticizer and contributed to improving mechanical properties and reducing moisture content and water solubility of the films	[117]
	Cinnamaldehyde in cassava starch/PLA bilayer film	Cinnamaldehyde-loaded cassava starch/PLA bilayer films exhibited antimicrobial properties against *E. coli* and *L. innocua*	[118]
Antioxidant action	Beetroot powder in starch-based hydrophobic bio-elastomer film	Betanin from beetroot was very effective in scavenging free radicals against 2,2-diphenyl-1-picrylhydrazyl free radical (DPPH) and 2,2′-azinobis(3-ethylbenzothiazoline-6-sulfonic acid) radical cation (ABTS+). Betanin was released by diffusion without disintegration of the polymer matrix. Beetroot powder incorporation could increase Young’s modulus of the bio-elastomers	[119]
	Cassava starch film with green tea extract and palm oil colorant	Antioxidant properties of polyphenols from green tea extract and carotenoid colorant from oil palm added antioxidant properties to the film. High concentrations of green tea extract should be avoided since the high content of polyphenols in green tea extract can be acted as a pro-oxidant agent. The films with antioxidant properties were effective for the storage of fatty foods. The incorporation of colorant and green tea extract could improve the mechanical and water vapor barrier properties of the films.	[120]
	Green tea extract in potato starch-based films	The films have improved antioxidant, water vapor barrier, mechanical, and thermal properties. The films showed great potential for the development of active antioxidant packaging for fresh beef.	[121]
	Curcumin (from *Curcuma longa* L.) in proso millet starch-based film	The phenolic compounds in curcumin imparted antioxidant properties to the film, but at the expense of film tensile strength. The addition of Curcumin could enhance the water and UV–visible light barrier properties of the films.	[122]
	Mango (*Mangifera indica* L.) and acerola (Malpighia emarginata DC.) pulps in cassava starch-based films	Fruit pulp incorporated into cassava starch-based films makes the films effective for packaging lipid-rich foods. Fruit pulps with high vitamin content should be avoided since vitamin C can act as a pro-oxidant.	[123]
	Rosemary extracts in cassava starch films	The polyphenols from rosemary extracts imparted an increase in their antioxidant activity to the films. The films having a high extract content showed better UV barrier properties. However, the presence of Rosemary extracts inhibited the formation of bonds between glycerol and starch molecules which negatively affected the water vapor permeability and mechanical properties of the films.	[124]
Antioxidant and antimicrobial action	Alcoholic extract of red propolis in cassava starch films plasticized with glycerol and reinforced with cellulose nanocrystals	The films showed effectiveness against coagulase-positive staphylococci in cheese and could slow the oxidation of butter. The antimicrobial and antioxidant properties of alcoholic red propolis were due to the presence of phenolic compounds	[125]
	Thymus kotschyanus essential oil in starch-chitosan composite film	Monoterpene phenols, especially thymol and carvacrol, in *T. kotschyanus* essential oil play a vital role in imparting antibacterial and antioxidant properties. Films show an inhibitory effect against *L. monocytogenes*, *E. coli* O157:H7, *S. aureus*, and *S. typhimurium*, while starch-chitosan films without essential oil show no effectiveness against *S. typhimurium.*	[126]
	Pinhão starch and citric pectin packaging film, functionalized with feijoa peel flour (a byproduct from Acca sellowiana)	The packaging contains bioactive compounds such as phenolics and flavonoids, imparting both antioxidant and antimicrobial properties. The films showed inhibitory effects against *E. coli*, *S. typhimurium*, and *Pseudomonas aeruginosa* and showed effectiveness in maintaining the quality of apples during storage, with no weight loss after 5 days of storage.	[127]

## 6. Potential of Utilizing Genetic Engineering Approaches to Tailor Starch for Bioplastics Applications 

The native starch from many plants lacks the necessary properties for direct industrial applications. Therefore, modification of starch by physical or/and chemical approaches is a common practice. In recent years, producing tailor-made starches within the growing plant has attracted much attention, as it would eliminate or reduce the need for post-harvest physical and chemical modifications of starch, saving money and the environment. *Inplanta* modification of starch opens new possibilities to enhance the positive attributes and eliminate the shortcomings of native starch in industrial applications. 

In this research area, *inplanta* modification of starch composition and molecular structure has become one of the major focuses since starch composition is crucial in determining subsequent applications of starch. For example, in packaging applications, high-amylose starch is preferred over high-amylopectin or normal starch. Amylose films have better mechanical properties and barrier properties than amylopectin films [128], with one study showing a more brittle nature and tensile failure of amylopectin films compared with amylose films [129]. Because of this, considerable attempts have been made to develop amylose-only [130,131] or amylose-rich starches [132] for use in bioplastics applications. Amylose is reported to be capable of forming strong films, due to more stable and stronger molecular orientation with essentially linear molecules [128]. 

Amylose-only barley starch (99% amylose-like α-glucan), produced by silencing all three genes of the starch branching enzymes using the RNAi technique, has been demonstrated to be a useful raw material for bioplastics production [131]. The extruded amylose-only starch prototypes were characterized by high stress and strain at break compared with the parental control starch prototypes, while the permeability to gas in the amylose-only starch prototypes was comparable to that in commercial Mater-Bi^©^ plastic [131]. The amylose-only starch had a significantly increased amount of lipids in the form of free fatty acids and phospholipids compared to normal starch [131]. A higher amount of lipids is likely to have positive impacts on the material properties of the amylose-only starch compared to normal starch. Overall, that study demonstrated the feasibility of utilizing biotechnological approaches to produce *inplanta* modified starches as a functional alternative to commercial plastics [131].

High-amylose potato starches produced by RNAi technology possess superior tensile and oxygen barrier properties compared to wild-type starch [132]. This is mainly due to the high amylose content together with altered molecular structure, with an increased proportion of the longest chain amylopectin. Hence, the high-amylose potato starch produced by RNAi technology could be of interest for industrial applications in producing films or coatings, without further physical or chemical modifications. That study demonstrated the possibility of modifying the starch molecular structure to produce *inplanta* modified starches, with favorable properties for bioplastics applications [132]. 

Starch granule size influences film properties such as film thickness, tensile strength, and elongation rate, with starch granules of smaller size being preferable [18]. Hence, *inplanta* modification of granule size to produce a new type of starch has been attempted, through the expression of a tandem starch-binding domain derived from *Bacillus* cyclodextrin glycosyltransferase in potato plants [133]. A novel type of starch with a mean granule size of 7.8 µm was produced (compared with 15.2 µm for control starch), while keeping the other starch properties unaltered, indicating the potential for using this novel type of potato starch for producing biodegradable plastic films [133].

Phosphorylation of starch has significant importance in various technical applications and potato starch has a relatively high content of phosphate esters compared to cereal starches. *Inplanta* modification of potato starch phosphate content for bioplastics applications has recently been reported in a study where a novel type of potato starch with low phosphate content obtained from transgenic potato was found to display high robustness, transparency, mechanical strength, and extensibility of films, even in wet conditions [134]. These beneficial attributes of films made from low-phosphate potato starch demonstrate the possibility of utilizing native starch without any post-harvest modification to produce films with desirable qualities. The wet performance of films with low phosphate content (2 nmol G6P/mg starch) could be due to the low phosphate content promoting hydration and amorphization of starch, without disrupting inter-chain interactions [134]. This cannot be achieved with starch with high phosphate concentrations due to the risk of breaking molecular junctions between starch molecules, resulting in the dissolution of the starch network [134]. 

## 7. Applications of Starch-Based Packaging Materials

Even though the research in the field of starch-based food packaging is at its early stage, there is a growing interest in using starch as a “green” natural resource for packaging [135]. The reasons behind this drive could be the low cost, abundance, and excellent film-forming properties of starch as discussed before in this review. As stated by one of the manufacturers of sustainable packaging, bioplastics have the potential to be cheaper in long run compared to conventional oil-based plastics [136]. Major forms of starch in packaging applications include starch-based coatings or starch-based films [135,137,138,139]. A coating differs from a film by the fact that a coating is directly applied on a food surface while a film is a stand-alone wrapping material [140]. The major drawbacks in formulating starch-based materials in blends and composites and the strategies to overcome those drawbacks have been discussed before in this review. Therefore, this section of the review will focus on the practical applications of starch-based packaging materials. 

Starch-based packaging materials are currently in use in both rigid packaging and flexible packaging. Rigid packaging applications accounts for around 45.6% of the global bioplastic market share in 2020 [141]. The techniques associated with processing starch-based materials into packaging applications can be divided into two categories; traditional process techniques (e.g., Extrusion, Foaming Processing, Film Casting) and emerging technologies (e.g., Electrospinning, Forcespinning, 3D-Printing, Reactive Extrusion) [142]. Some example products of commercially available starch-based packaging raw materials, their ingredients, and their potential applications are summarized in Table 3. 

## 8. Future Perspectives and Outlook 

Future research should examine the potential for using starch in higher amounts for starch-based blends and composites for bioplastics applications, to produce highly biodegradable materials from this abundantly available natural resource. It could also examine the possibility of using starch as the sole polymer in the formulation of bioplastic materials. Importantly, future research should also investigate possible approaches to address the major drawbacks of starch-based bioplastics, such as high-water sensitivity and poor mechanical properties. Apart from looking for chemical methods to compatibilize starch and other polymers in blends and composites, efforts should be made to develop methods that are more sustainable for both the environment and the economy. In addition, *inplanta* modification of starch using novel genetic engineering approaches might prove an important area for future research, to eliminate the need for post-harvest physical and chemical modification of starch for bioplastics applications, particularly for the packaging sector. 

## 9. Conclusions

Applications of starch as a bioplastics material, particularly for use in the packaging sector, have been extensively researched. Starch has been shown to be a promising biopolymer with great potential in bioplastics applications. Starch is used in blends with both fossil fuel and other bio-based polymers to facilitate the degradability of the synthetic polymers, reduce the cost, achieve better mechanical properties for subsequent applications, and minimize the problems associated with starch (e.g., water sensitivity, poor mechanical and barrier properties). However, incompatibilities between starch and other polymers need to be addressed by an appropriate selection of approaches to improve the compatibility between the polymers. Modification of starch and/or other polymer components, using plasticizers, reinforcing agents, and compatibilizers have shown positive results in minimizing the problems associated with the incompatibility of starch and other polymers when used as a bioplastics packaging material. Apart from the post-harvest modifications of starch, in-planta modification of starch has resulted in the generation of tailored starches that are better suited for bioplastics applications. These approaches are also more sustainable, since they potentially reduce or minimize the need for post-harvest modification of starch or minimize the requirement of a second polymer, compatibilizers, reinforcing agents, etc. in bioplastics applications. Apart from the biodegradability and other mentioned advantages of starch-based materials, starch-based composites with antimicrobial and/or antioxidant properties, for food packaging applications, have attracted research interest in recent years as packaging materials with added advantages to ensure food safety.

## Figures and Tables

**Table 1:** Types of fibers used as reinforcing agents to produce thermoplastic starch (TPS).

Type of Fiber	Type of Starch Used in TPS Production	Type of Plasticizer Used	Remarks	Important Results	Reference
Barley straw	Potato starch	Glycerol	A Twin Screw Extruder was used to plasticize starch. Mixing TPS with fibers was done in a rheometer at 100 °C and with screws speed at 120 rpm	Barley straw fiber closely adhered to the TPS matrix. Rigidity and strength of the TPS matrix significantly enhanced when 15% (by weight) of barley straw fibers was used	[49]
Grape waste	Potato starch	Glycerol	A Twin Screw Extruder was used to plasticize starch. Mixing TPS with fibers was done in a rheometer at 100 °C and with screws speed at 120 rpm	Grape waste fiber poorly adhered to the TPS, and the addition of grape waste fiber did not contribute to enhancing the rigidity and strength of the TPS matrix	[49]
Oil palm mesocarp	Cassava starch	Glycerol	Composites of TPS and oil palm mesocarp fibers were prepared using a screw extrusion rheometer. Both raw and alkaline treated oil palm fibers were tested for the properties of the composites.	Significant improvement in elastic modulus maximum stress and thermal properties of the composites with raw fiber were reported due to presence of silica in the fiber which interacts with OH groups in TPS. The optimal results for mechanical properties were displayed at 10 wt% of the raw fiber inclusion level.	[50]
Cellulose from rice husks and coffee husks	Corn starch	Glycerol	Composites were prepared by melt blending in a two-roll mill and both types of fibers were tested at 1 wt%, 5 wt%, and 10 wt% levels	Both types of fibers contributed to improving film stiffness but at the cost of film stretchability. Coffee husk fibers were reported to maintain the film ductility at 1 and 5 wt% levels. Rise husk fibers at any incorporation level and coffee husk fiber at the highest incorporation level were effective in reducing the oxygen permeability of composite films.	[51]
Cogon grass	Cassava starch	Glycerol	Composites were prepared using compression molding. Cogon grass fiber was tested at 1, 3, and 5 wt% incorporation levels	Good adhesion of cogon grass fiber with the TPS matrix was observed. The incorporation of Cogon grass fiber improved the tensile strength, flexural properties, and water resistance of the composites and, reduced elongation at break, impact stress, and thermal properties.	[52]
Sugar palm	sugar palm starch	Glycerol	Composites were prepared using hot pressing in a Carver hydraulic hot press. Sugar palm fibers were tested at 0, 10, 20, and 30 wt% incorporation levels.	Better interfacial bonding between fiber and matrix was observed and the mechanical (tensile and flexural) properties and thermal stability of the composites improved with the incorporation of sugar palm fibers. Water uptake and moisture content of the composites decreased with the incorporation of fibers.	[53]
Banana leaf (BLF)	Corn starch	Glycerol	10 wt% beeswax was also included for the mixture of 63 wt% starch and 27 wt% glycerol when preparing the thermoplastic cassava starch. The BLF content was varied from 10 to 50 wt% when preparing the TPCS/BLF composites	Increments in the mechanical (tensile and flexural) properties and thermal stability were observed when BLF was incorporated. The highest strength and modulus values of the material were reported at 40 wt% BLF content	[54]
Cassava bagasse	cassava starch	Glycerol	Films were prepared by solution casting and cassava bagasse lignocellulose fiber suspension was tested in two concentrations (0.65% and 1.3%, *w*/*w* and compared with nanoclay suspension of the same concentrations (0.65% and 1.3%, *w*/*w*).	Films reinforced with cassava bagasse lignocellulose fiber showed increased tensile stress and reduced elongation at break value, lower water vapor permeability, and thermal stability compared to films reinforced with commercial-grade nanoclay	[55]

**Table 3:** Examples of market available starch-based packaging materials.

Product/Brand Name	Type of Starch and Other Ingredients	Potential Applications	Manufacture and Reference
Bioplast^®^ 300	Potato starch and other biologically sourced polymers	Suitable for blown-film extrusion applications (e.g., Bags for fruit and vegetables and films like mailing films)	BIOTEC biologische Naturverpackungen GmbH & Co., Germany [143,144]
BIOPLAST^®^ 400	Potato starch and other biologically sourced polymers	Suitable for blown-film extrusion applications (e.g., Bags for fruit and vegetables and films like mailing films)	BIOTEC biologische Naturverpackungen GmbH & Co., Germany [143,144]
BIOPLAST^®^ 500	Potato starch and other biologically sourced polymers	suitable for blown film extrusion applications, especially light films with a thickness of approx. 15 μm (e.g., Waste bags)	BIOTEC biologische Naturverpackungen GmbH & Co., Germany [143,144]
BIOPLAST^®^ GF 106/02	Potato starch	suitable for blown film extrusion applications	BIOTEC biologische Naturverpackungen GmbH & Co., Germany [143,144]
Terratek^®^ SC50	Wheat starch (50% by weight) and polypropylene	Suitable for injection molding applications	Green Dot Bioplastics, United States [145]
Terratek^®^ SC65	Wheat starch (65% by weight) and polypropylene	Suitable for injection molding applications	Green Dot Bioplastics, United States [145]
Terratek^®^ SC200012	starch and bio based polyethylene	Injection molding applications	Green Dot Bioplastics, United States [145]
Terratek^®^ SC200041	starch and bio based polyethylene	Injection molding applications	Green Dot Bioplastics, United States [145]
PaperFoam^®^	Potato starch and other biobased ingredients	Injection molding applications (e.g., egg cartoons)	PaperFoam, The Netherlands [146]
Mater-Bi^®^ types (e.g., Mater-Bi^®^ NF803 (grade N)	Composition varies on the type (e.g., Mater-Bi^®^ NF803 (grade N) contains a starch-based fraction, a synthetic biodegradable polyester, and additives)	Filming, extrusion, thermoforming and injection applications	Novamont, Italy [147,148]
Solanyl^®^	side stream starch from the potato processing industry and/or grain, root or seed flour-based resources	Injection molding, sheet and profile extrusion, thermoforming and extrusion film casting or blowing products and processes	Rodenburg Biopolymers, The Netherlands [149]

## Data Availability

Not applicable.
